# Challenges and Opportunities in Scaling-Up Nutrition in Healthcare

**DOI:** 10.3390/healthcare3010003

**Published:** 2015-01-09

**Authors:** Ian Darnton-Hill, Samir Samman

**Affiliations:** 1 The Boden Institute of Obesity, Nutrition, Exercise & Eating Disorders, Faculty of Medicine, University of Sydney, Sydney, NSW 2006, Australia; 2 Gerald J. and Dorothy R. Friedman School of Nutrition Science and Policy, Tufts University, Boston, MA 021111, USA; 3 Department of Human Nutrition, University of Otago, PO Box 56, Dunedin 9054, New Zealand; E-Mail: samir.samman@otago.ac.nz; 4 Discipline of Nutrition and Metabolism, School of Molecular Bioscience, University of Sydney, Sydney, NSW 2006, Australia

**Keywords:** healthcare, capacity, environment, access, low- and middle-income countries

## Abstract

Healthcare continues to be in a state of flux; conventionally, this provides opportunities and challenges. The opportunities include technological breakthroughs, improved economies and increasing availability of healthcare. On the other hand, economic disparities are increasing and leading to differing accessibility to healthcare, including within affluent countries. Nutrition has received an increase in attention and resources in recent decades, a lot of it stimulated by the rise in obesity, type 2 diabetes mellitus and hypertension. An increase in ageing populations also has meant increased interest in nutrition-related chronic diseases. In many middle-income countries, there has been an increase in the double burden of malnutrition with undernourished children and overweight/obese parents and adolescents. In low-income countries, an increased evidence base has allowed scaling-up of interventions to address under-nutrition, both nutrition-specific and nutrition-sensitive interventions. Immediate barriers (institutional, structural and biological) and longer-term barriers (staffing shortages where most needed and environmental impacts on health) are discussed. Significant barriers remain for the near universal access to healthcare, especially for those who are socio-economically disadvantaged, geographically isolated, living in war zones or where environmental damage has taken place. However, these barriers are increasingly being recognized, and efforts are being made to address them. The paper aims to take a broad view that identifies and then comments on the many social, political and scientific factors affecting the achievement of improved nutrition through healthcare.

## 1. Introduction

Nutrition has infrequently played a major role in healthcare, despite the anecdotal observation that most people perceive that it should be an important part of any care. It has tended to be implemented mainly for mother and child health and nutrition. The professions devoted to nutrition and diet in healthcare systems have tended to be of less status and have less power than medical care personnel. This appears to be true even in countries where under-nutrition is a major healthcare issue. With the epidemic of being overweight and obesity and the nutritional-related non-communicable diseases (NCD), this is changing. This commentary aims to examine the changes that are happening and reviews both the existing challenges to increasing the role of nutritional prevention and management in healthcare and some of the opportunities to address the changing patterns. We take a broad view of the many social, political and scientific factors that affect nutrition and health and, hence, the need for healthcare to take such factors into account.

## 2. Background

Healthcare continues to be in a state of flux, which conventionally should provide both opportunities and challenges. Healthcare overall has improved for most populations over the last century, and life expectancies have improved in most countries [1]. In 2012, life expectancy at birth for both sexes globally was 70 years, having increased by an average of six years since 1990 [1]. In Japan or Sweden, a child can expect to live more than 80 years; in Brazil, 72 years; India, 63 years; and in one of several African countries, fewer than 50 years [2]. These trends have occurred both for reasons of improved healthcare and because of economic growth and social and cultural change. These changes have included improved and safer diets. On the other hand, the increased consumption of highly processed foods and the increased consumption of saturated fats and refined carbohydrates have led to an epidemic of chronic diseases [3]. Within countries, life expectancy is much lower in the poorest of the poor, because of excess levels of illness and premature mortality. However, in all countries, health and illness follow a social gradient: the lower the socioeconomic position, the worse the health [2,4], and this is true also of chronic diseases [4,5]. This narrative review will address the aim above, using some of the global data available, by identifying the challenges, especially with relation to the nutritional aspects of healthcare, and then the opportunities that might improve nutritional outcomes in healthcare.

There has long been a recognition, if more rarely sufficient action, of hospital malnutrition, particularly in aged care and surgical care. However, the wider implications of malnutrition (both under- and over-) have been more recent, especially in healthcare. The recent attention to under-nutrition in maternal, infant and young care in health and developmental outcomes has marked a paradigm shift in the approaches in low- and middle-income countries (LMIC) and a welcome shift in attention to including maternal and adolescent health and nutrition [6,7,8]. In 2011, there were an estimated 101 million children under five years of age who were underweight, but this was down from an estimated 159 million in 1990 [9]. While under-nutrition levels have slowly decreased, although unequally across countries and regions, the levels of adults and children being overweight and obese continues to increase at alarming rates, the so-called ‘double burden of disease’ [10]. Therefore, while many indicators, such as life expectancy, declining rates of under-nutrition and micronutrient deficiencies, maternal nutrition and mortality, under-five mortality rates, and so on, have all improved, they remain highly inequitable across regions and countries, and many challenges remain.

## 3. Global Shifts

NCD now make up the largest contribution to mortality, both globally, as well as in the majority of LMIC [10,11]. At the same time, food and nutrition insecurity continues to affect over 800 million people globally [3]. Worldwide, NCD account for 60% (35 million) of global deaths [12]. The largest—80% (28 million)—occurs in LMIC, making NCD a major cause of poverty and an urgent development issue [13]. NCD, including the more directly nutritional-related chronic diseases, will be the leading global cause of disability by 2030 [1]. The greater number will be in LMIC, where eight million people below the age of 60 years die each year from preventable causes, including unhealthy diets, tobacco use, alcohol consumption and physical inactivity [1,12]. From a nutrition-in-healthcare perspective, the metabolic syndrome spectrum is made up of risk factors and metabolic disorders that will respond to healthcare management and lifestyle modifications, including the effects of diet and physical exercise [14]. At a minimum, metabolic syndrome includes dyslipidemia, elevated glucose, hypertension and obesity [15] (Table 1), all of which have implications for healthcare and healthcare costs, both individual syndromes and the cumulative effect on incident type 2 diabetes mellitus and cardiovascular diseases (CVD) [14,16].

Lack of access to affordable medicines and healthcare services are also major causes of these preventable deaths. Globally, the NCD burden is estimated to increase by 17% in the next ten years and in the African region by 27%. The highest absolute number of deaths will be in the Western Pacific and South-East Asia regions [1]. In the USA, the lifetime risk of diagnosed diabetes mellitus from age 20 years on is 40% for men and women, representing increases of 20 and 13 percent, respectively, since 1985–1989 [17]. The highest lifetime risks were in Hispanic and African-American populations, again reflecting the socio-economic and lifestyle disadvantage, although there does appear to be ethnic susceptibilities, as well, e.g., higher levels of hypertension in African-Americans. The global prevalence of diabetes mellitus was 8.3% in 2011 and is expected to increase to 9.9% in 2030 among adults aged 20–79 years old [18].

This increase in prevalence, coupled with inflation, has contributed significantly to the increase in economic costs attributable to individual components of metabolic syndrome, such as diabetes mellitus and overall healthcare utilization and costs [16,17]. With consequent rising healthcare costs, this rapidly changing health and disease profile has serious implications for poverty reduction and economic development. Low-income countries that are still grappling with heavy burdens of infectious disease risk and serious levels of under-nutrition are being overwhelmed by this rise of largely preventable NCD. In India, for instance, the projected foregone national income due to NCD, between 2005 and 2015, has been estimated to be $US 236.6 billion [5]. The estimated cumulative economic losses to LMIC from the four main NCD (not all directly nutritionally-related) are estimated to surpass US$ 7 trillion over the period 2011–2025 (an average of nearly US$ 500 billion per year) [5]. This yearly loss is equivalent to approximately 4% of the countries’ current annual output, and the report notes that one factor contributing to this macroeconomic impact is that almost 30% of NCD-related deaths in LMIC occur in people under 60 years of age [5]. The calculated costs of NCD in countries like Australia, the U.K. and the USA are in the many millions.

**Table 1:** Definition of metabolic syndrome ^1^.

Risk Factor	Country/Ethnicity	Gender	
		Male	Female
Waist circumference (cm)	Europids; Sub-Saharan Africans; Eastern Mediterranean and Arab populations	≥94	≥80
	South Asians; Chinese; Japanese; South and Central Americans	≥90	≥80
Plasma triglycerides mmol/L		≥1.7	≥1.7
Plasma high density lipoprotein cholesterol mmol/L		≤1.03	≤1.29
Blood pressure mm Hg		≥130 (systolic), ≥85 (diastolic)	≥130 (systolic), ≥85 (diastolic)
Fasting plasma glucose mmol/L		≥5.6	≥5.6

^1^ According to the International Diabetes Federation, for a person to be defined as having metabolic syndrome, they must have central obesity, defined as a waist circumference as indicated above [15].

Analogous increases occur in all countries, e.g., diabetes mellitus is calculated to have cost the Australian health system over $AU 1.5 million in 2008–2009, an increase of 86% since 2000–2001 [9,19]. Data from the Australian Diabetes, Obesity and Lifestyle Study, collected in 1999–2000 and 2004–2005, showed direct care costs alone were $AU 21 billion and consisted of ambulance services, hospital visits, prescription medication and items, such as blood glucose self-monitoring meters and strips [19,20]. In Singapore, diabetes mellitus is one of the top ten causes of death and its prevalence among adults is projected to increase from 11.3% in 2010 to 15% in 2050 with consequent increased health financing required [21]. A World Economic Forum report by Harvard University estimated that for CVD, chronic respiratory disease, cancer, diabetes mellitus and mental health, there would be a cumulative output loss of US$ 47 trillion over the next two decades [11]. This loss represented 75% of global GDP in 2010 (US$ 63 trillion). While monetary figures in the trillions are hard to grasp, they helpfully point out that this also represents enough money to eradicate two-dollars-a-day poverty among 2.5 billion people for more than half a century [11]. Nearly all nations also have increasingly large ageing populations, including in countries with low access and low capacity and already overburdened with children who are underweight, high maternal mortality rates and infectious disease burdens from tuberculosis, malaria and AIDS. The impacts are especially severe on countries where there are already insufficient funds and a low healthcare capacity to deal with existing high levels of under-nutrition and infectious diseases. These rising costs represent an important global challenge.

## 4. Barriers to Healthcare

**Figure 1:**
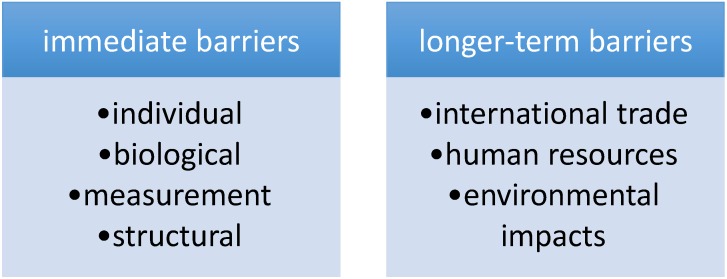
Immediate and longer-term barriers to healthcare.

Immediate barriers, individual, biological, measurement and structural, are discussed, as are longer-term barriers, such as international trade, staffing shortages where most needed and environmental impacts on health (Figure 1). The subsequent Section 5 outlines how some of these challenges may be addressed.

### 4.1. Immediate Barriers

#### 4.1.1. Individual

The need for healthcare and increased attention to nutrition as part of that healthcare is a reflection of the major causes of ill health globally today. Individual factors contributing to ill health not being addressed adequately can be a barrier to optimizing health, e.g., the risk factors for metabolic syndrome as outlined above. Feeding patterns and poor sanitation patterns both contribute to under-nutrition in young children, amongst other factors, such as inadequate care and concurrent infectious diseases. There is a need for a wider view of individual determinants that healthcare needs to address. Conversely, over-attention to individual factors can lead to insufficient attention to environmental, social and political factors acting against the more optimal nutrition of an individual in today’s society [22]. Whereas the population numbers of those who are food and nutrition insecure have not much changed (around 800 million globally), the numbers of over-nourished have increased dramatically: a third of all children now are overweight or obese in some major economies [9]. The numbers continue to increase in virtually all countries. The prevalence of nutrition-related NCD, such as type 2 diabetes mellitus, has increased dramatically, even in countries, such as China, which traditionally had very low levels. The three leading causes of years of life lost due to premature mortality are coronary heart diseases, lower respiratory tract infections, such as pneumonia and stroke [12], and those nutrition-related NCD continue to rise.

On the positive side, a recent study using four different indices of healthy diets found convincing evidence of higher diet quality being associated with decreased risk of all-cause, CVD and cancer mortality among older adults [23]. Those people who ate the healthiest diets at the average age of 50 also had an almost 9% lower risk for dementia in a 14-year follow-up study compared with those whose diets were least healthy [24].

The emphasis on the individual to change their unhealthy habits has been the approach by the food and drink industry. Evidence is accumulating that the results, especially for weight loss, of this approach have not been very successful [25], although a recent meta-analysis showed a small, but significant, benefit (in obese patients) with behavioural interventions involving both diet and physical activity [26]. Increasingly, it is being accepted that there must also be a conducive and supportive environment [27]. There is good and accumulating evidence that high consumption of sweetened beverages is associated with increased risk of being overweight in children and NCD in adults [28,29,30]. While at some level, individuals do need to change their behaviours to ones recognized as healthier, such as balanced diets and more exercise, it is increasingly clear that this is not so easy, especially in terms of losing excess weight and sustaining it [25]. This is most successful when supported by informed healthcare personnel supported by legislation and social changes [22,27]. Environmental changes, such as making walking safer and more accessible and reducing the ubiquity of unhealthy foods, e.g., at schools, are receiving increasing support, but not without resistance from vested interests [31].

#### 4.1.2. Biological: Epigenetic Effects of Poor Nutrition

It is a well-known observation that overweight adults tend to have overweight children (and pets), emphasizing the role of behavioural and familial factors. What is also now clear is that genetic programming occurs in the foetus during pregnancy, such that the infant carries a metabolic ‘load’ already by birth that can predispose it to diseases, such as obesity, diabetes mellitus and CVD [32]. Of even more concern is that these epigenetic changes not only persist into adulthood, but also are transmitted into the next generation [33]. These factors represent a barrier in that, while widely accepted, current healthcare is not well equipped to address. As they are intergenerational, yet again, social, political and environmental interventions are likely to be needed.

A study from Finland and Sweden was able to demonstrate a genetic risk score based on 13 single nucleotide polymorphisms (SNP) associated with CVD and a roughly 70% increase in risk for affected individuals (about 20% in their study) [34]. The increasing uses of genetic risk scores in healthcare will likely increase costs even more and impact on healthcare systems. It will also likely be mainly for those who can afford ‘personalized medicine’ and help increase the socioeconomic gap between countries and within countries.

The review by Sim [33] noted that an already complex set of influences is being further complicated by social factors, such as an increasing trend to later childbearing coupled with the rising prevalence of parental obesity. Maternal obesity is related to an increased risk of NCD and all-cause mortality in the offspring, usually presenting in adolescence and adulthood [35]. Therefore, pre-pregnancy and gestational obesity may lead to a self-reinforcing vicious cycle of excessive weight gain and adiposity that is passed on from mother to offspring. More recent studies have also reported adverse outcomes associated with an obese biological father, as well as the mother [36].

It is striking that the earlier historic ‘vicious cycle’ was the one related to under-nutrition and infection, whereby infectious diseases were made worse by under-nutrition and the disease made the under-nutrition worse by increasing need, reducing feeding and absorption, and so on. A more recent recognition was that stunted women have small babies who, in turn, give birth to stunted babies, and so, an intergenerational vicious cycle is also at play. It is now recognized that these same low birth weight babies, if they later gain weight, such as has happened in much of India and other middle-income countries, the risk of NCD later in life are greatly enhanced [32]. These cycles must be interrupted by accessible healthcare, attention to nutrition and socially supportive measures.

#### 4.1.3. Measurement: Increasing the Evidence Base for Effectiveness

**Figure 2:**
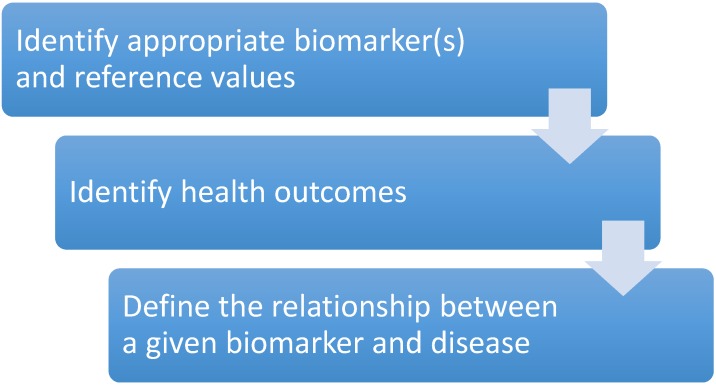
Challenges in monitoring and evaluation.

The ability to assess the role of nutrition in healthcare is underpinned by the availability of reliable biomarkers that reflect nutrient status, as well as those used in operational monitoring. Appropriate biomarkers are essential for the assessment of the impact of the nutrition on the health status of the target population and lead to improved monitoring and evaluation. Biomarkers serve as a key element in any monitoring and evaluation programme, which is increasingly becoming a core aspect of nutrition interventions. Screening with routine biomarkers, such as blood glucose or serum cholesterol concentrations, to determine the risk of metabolic syndrome and its components [15] (Table 1) is based on the premise that timely treatment may prevent or delay the onset of complications [37]. Usually, the preliminary screening is followed-up by further testing in high-risk individuals as a routine part of good healthcare. Established biomarkers, such as glucose and cholesterol, require rudimentary technical skills and often are carried out by allied health professionals who are not necessarily technicians. However, the identification and use of biomarkers for micronutrient deficiencies is technically challenging and often requires advanced technical skills, agreement on the choice of biomarkers and harmonisation of reference values (Figure 2). To this end, international initiatives around the provision of reliable data on biomarkers of micronutrients have been established recently, namely the National Institutes of Health Biomarkers of Nutritional and Development (BOND) initiative [38] and the WHO electronic Library of Evidence for Nutrition Actions (eLENA) [39]. Both resources highlight the challenges in the identification of biomarkers for nutrition and development.

BOND has established a “query-based system” around biomarkers for use by researchers, clinicians, programme/intervention agencies and policy makers. This system is intended to provide data to researchers, clinicians and the agencies that fund them, using the most appropriate biomarkers. The purpose of this approach is to improve intervention study designs and instil confidence in the interpretation of the outcome data [40]. WHO, through the eLENA resource, provides a point of reference for nutrition guidelines, recommendations and related information and supporting material, such as background documents and commentaries. eLENA aims to help programme leaders and policy makers with implementing and scaling-up nutrition interventions [39].

Challenges remain in the identification of appropriate biomarkers with surprisingly many gaps in our knowledge of factors that are likely to impact biomarkers, such as the magnitude of body stores, utilization, responsiveness to changes in intake and the many disparate functions of most nutrients. Confounding factors unrelated to the nutrient of interest, such as sex, age and genotype, are becoming increasingly important. In particular, inflammation, either as chronic low-grade or acute inflammation, is recognised increasingly as an important confounder in the interpretation of many biomarkers of nutritional status [38,41]. The use of multiple biomarkers should theoretically increase the reliability of the assessment of status by removing the influence of confounders, such as inflammation. In so doing, it may be appropriate to consider biomarkers that are not directly related to the primary biomarker, such as anthropometric measurements and/or measures of dietary intake. Equally, this includes a biomarker’s ability to detect the risk of toxicity when the intervention programme has unexpected increases due to the dose or the presence of hyper-responders within the population [38].

Healthcare providers and human resources for health also require guidance on logistic factors that are associated with effective monitoring and evaluation. At the local level, this may include both organizational measures, as well as the very practical aspects, such as the collection and short-term storage of samples, which add to the variability in the reported outcome data. It is essential to know, or at least to harmonize the conditions for sample collection and subsequent handling. In many LMIC, health centre samples need be transported to advanced laboratories for subsequent analysis, with the added complication of shipping and longer-term storage. This aspect of planning is important for all stakeholders, given the relatively high costs involved and, importantly, for the application of the findings to local healthcare. Capacity building in personnel that are involved in monitoring and evaluation is discussed below.

#### 4.1.4. Structural

The social determinants of health are the conditions in which people are born, grow, live, work and age. They determine both the nutritional and health wellbeing of populations and are shaped by the distribution of money, power and resources at global, national and local levels [4]. The social determinants of health are mostly responsible for health inequities: the unfair and avoidable differences in health status seen within and between countries [4,42]. Such determinants are often expressed through nutritional outcomes, both under- and over-nutrition and their consequences. Given the widespread acceptance of the influence of ‘obesogenic environments’, the epidemics of being overweight and obesity could well be considered structural outcomes of the way food changes have been made, ubiquitous advertising and increased portion sizes, as well as changes in physical activity. Institutional and structural barriers include negative impacts of globalized trade and environmental factors, such as the ubiquitous ‘obesogenic environment’ [22]. Part of this is the wide availability of sugar-sweetened soft drinks to schoolchildren [43], and their relationship to weight gain is now firmly established [44]. WHO, amongst other authorities, now have guidelines for Member States to be used in controlling the advertising of unhealthy foods to children, especially on television.

Therefore, while life expectancy has, on average, increased around the world, it has been unequal, ranging from Sierra Leone (46 year life expectancy at birth) to women in Japan at 87 years [12]. At the same time, there have been rises in childhood obesity, with 44 million children under five years of age (6.7%) being overweight or obese in 2012 (up from five percent in 1990). A concerning aspect of the future trends is that 10 million of the total 44 million overweight or obese children live in Africa [12]. These are trends in nutrition that healthcare will need to address more directly, as well as through ‘nutrition-sensitive’ interventions [6,8] and social and political measures [22].

### 4.2. Longer Term Barriers

#### 4.2.1. International Trade

While it is generally accepted that globalization has raised the standard of living for probably the majority of people globally and, therefore, no doubt contributed to the average life expectancy increasing, it has also contributed in a major way to the rise in being overweight and obesity and the nutrition-related NCD. Many of the existing international trade practices can be counter-productive to LMIC, avoiding the nutrition-related NCD epidemic and especially practices to combat it, e.g., attempted actions against smoking and highly processed foods [31]. On the other hand, because the vast majority of people buy all or some of the food in their diets through the market systems, the industry will have to be part of any solutions. The inherent tension of these behaviours is currently being played out, so far with little success, with self-regulation likely to be insufficient [31,45]. More positive has been the private sector’s involvement in fortification, such as the spread of iodized salt. The provision of healthcare has in itself become part of international trade, with increasing hubs of healthcare tourism in centres, such as Bangkok, South Korea and elsewhere.

#### 4.2.2. Staffing Shortages Where Most Needed

Healthcare systems clearly cannot function without human resources for health, but increasingly, healthcare providers are migrating out of their home countries, especially from LMIC, to go to work in more affluent countries [46]. In 2006, WHO estimated that there was a shortage of more than 4.3 million health personnel across the world, with low-income countries being particularly affected [42]. Much of the flow of trained healthcare workers is to affluent countries that are failing to address shortages and vacancies of domestically-trained professionals [46], and as trade barriers come down, globalization is allowing the increased mobilization of professionals throughout the world, including healthcare professionals. The problem is predicted to increase [46]. Insufficient human resources for health in developing countries have been identified as one of the main constraints limiting progress on such initiatives, as the “3 by 5” initiative, which addresses NCD, and the Millennium Development Goals (MDG) [46].

The healthcare costs of the obesity pandemic have been mentioned. A McKinsey Report reported a WHO estimate of those in many developing countries, where obesity now accounts for two percent to seven percent of all healthcare spending. They also noted that medical costs are only a small fraction of the pandemic’s total costs [47]. Nevertheless, the costs to total healthcare of training and then retaining and paying salaries are considerable, especially in resource-constrained health systems, and so, shortages of physicians, nurses and other health personnel, such as dieticians and nutritionists, are currently ‘jeopardizing health systems advances’ in LMIC [48]. While WHO and others have attempted to address these human resource problems, a significant proportion of LMIC students still intend to work abroad or in cities after completion of training [48]. Health worker retention is also a problem in many high-income countries, but is currently being partially resolved by the encouragement of healthcare personnel to emigrate from LMIC [48]. This aggravates the situation, as these countries are already having their own shortages.

#### 4.2.3. Environmental Impacts on Global Health

One result of climate change leading to drought, altered disease patterns and rising demand by increasingly affluent middle-income country populations is the likely increase in the costs of food, as happened dramatically in 2007–2009, which led to, amongst other things, social unrest [49]. Recently, FAO has noted that many of the foods that many cultures consume for breakfast, such as coffee, orange juice, wheat products, sugar, milk butter, cocoa and pork, have all risen dramatically in price (almost 25% this year, 2014) [3]. This is already having repercussions in populations and markets, and further concerns are being expressed about El Niño weather patterns leading to extreme weather in many parts of the world. In the previous food prices global crisis, those most affected were the urban poor of food-importing countries [49]. The health risks, both present and future, from global climate change are increasingly clear and the evidence science-based, although action is excruciatingly slow [50]. There are both immediate and direct risks, e.g., from changing climate patterns of increased heat waves, extreme weather events and altered air quality; and indirect risks from changes and disruptions to ecological and biophysical systems, affecting food yields, the production of aeroallergens, bacterial growth rates, the range and activity of diseases vectors (such as mosquitoes) and water flows and quality [50]. The urgent need to reflect such changes in healthcare [51] and in nutrition security activities [52] needs to be part of any solution.

## 5. Opportunities

### 5.1. Improved Science and Technology to Increase Access to Healthcare

Against a background of improved science and technology, nutrition has received an increase in attention and resources in the last couple of decades, a lot of it stimulated by the rise in obesity and related diseases, such as type 2 diabetes mellitus and hypertension. As noted, ageing populations and rising healthcare costs of managing chronic diseases also have meant increased attention to nutrition-related chronic diseases and their prevention. In many middle-income countries, there has been an increase in the double burden of malnutrition with undernourished children and overweight/obese parents and adolescents. In low-income countries, there has been an increased evidence base established on how to address under-nutrition [6] and a concomitant increase in the proportion of aid funds going to scaling-up nutrition interventions, both nutrition-specific and nutrition-sensitive interventions. The most dramatic example has been the Lancet articles on maternal, infant and young child under-nutrition, with a welcome recent increased emphasis on adolescence, in 2008 and 2013. These series have established a large degree of consensus on which interventions are effective and how they should be scaled-up [53]. One of the many strengths of these two series has been the clear demonstration of economic benefit in nutritionally intervening in early childhood in terms of improved schooling, height and, later, earning capacity [7], along with improved healthcare, both for the pregnant woman and for the new infant. Along with many other reports, the series also documents the rise of young children being overweight and obese [8]. While it has undoubtedly influenced the increased attention to nutrition in healthcare and development, it has also been criticized as being too narrow for the real complexity of food and nutrition outcomes [54] beyond the health sector. The remaining challenges are to scale-up effectively and to build the evidence base for addressing the epidemics of obesity and overweight, including in children, and the associated NCD [5].

### 5.2. Strengthening the Nutrition Component of Healthcare and Medical Training

Given the increasing attention being given to nutrition causes and consequences as described above, there is a need to increase training capacity, including of nutrition professionals, and to improve the retention and management of the health and nutrition workforce [42]. Particularly in the medical profession, nutrition training has traditionally been inadequate and superficial, but there is some evidence of changing perceptions. Similarly, nutrition professionals have increased power within the health system, but still need good career path opportunities. Team approaches to the prevention and management of NCD are also opportunities for nutrition and dietetic professionals.

A McKinsey Report on obesity noted the “complex, interdependent relationships among the etiological factors and the challenges this makes for losing weight but also to healthcare professionals and by implication their training” [47]. The challenges of involving whole communities to address the multiple causes, including those beyond the normal attention of health and nutrition-care professionals, which appears to achieve the best results, is also a challenge with many different players, objectives and funding sources all needing to be somewhat coordinated [47]. Although there have been some shifts over the last quarter of a century in terms of increased nutrition training for medical doctors and nurses involved in front-line healthcare, there remains much to be done. Nutrition specialists, such as dietitians and public health nutritionists have been trained in increasing numbers. Certainly, the interest (and also access to sometimes spurious information) of the general public has greatly increased. Nevertheless, this is an area where a great deal needs to be done, while at the same time nutritional science is expanding all the time, and there is now a stronger evidence base for such healthcare advice, e.g., the now universally-accepted advice to eat more fruits and vegetables.

### 5.3. Structural Interventions to Increase Access

The access to, and quality of, healthcare have improved greatly, but these are still dramatically different across nations and populations. The gap between the most- and least-affluent populations has grown, not the least of which is because of poor access, lack of capacity, out-migration of healthcare personnel, insufficient funding, inequity and political systems [10]. The USA, the largest economy in the world, has some of the worst health outcomes of the OECD countries [42], again preferentially favouring the higher socio-economic strata. While improved healthcare has been an important part of longer life expectancies and reduced unnecessary morbidity and mortality, it is by itself insufficient without social change and better equity and attention to the social determinants of health [1], as frequently noted in this commentary.

It has been observed that governments—national, regional and local—must play ‘a uniquely powerful role’ and can catalyse change by offering incentives, helping align efforts, taxing unhealthy foods and legislating on advertising [47]. An important first step would be to make clear to all that the obesity pandemic is one of each government’s top priorities [47], and this is starting to happen. The World Health Assembly (WHA) has had endorsed by its Member States an NCD Strategy and 2008–2013 Action Plan for the Global Strategy for the Prevention and Control of NCD [55]. This outlines a working plan to prevent and control CVD, diabetes mellitus, cancers and chronic respiratory diseases. The four shared risk factors—tobacco use, physical inactivity, unhealthy diets and the harmful use of alcohol—are all being targeted [55].

It has been suggested that because the current approach is largely a high-risk individual approach, this approach may in fact widen socio-economic inequalities, as such approaches involve clinic visits and advice on lifestyle factors, such as diet and physical activity, and tablets to reduce cholesterol and blood pressure. Such inequalities have been reported in screening, healthy diet advice, statin and anti-hypertensive prescribing, adherence and smoking cessation [56]. Consequently, there are increasing calls for universal health coverage, particularly around the post-2015 goals [57]. In the meantime, individual healthcare measures should therefore be supplemented by population-wide NCD prevention, including dietary advice and other measures, such as legislation on advertising, especially for children, and the use of taxes on sugar-sweetened soft drinks and some foods [58]. There is increasing pressure, by, amongst others, the World Obesity Federation and Consumers International, to regulate unhealthy food in the same way that cigarettes are starting to be regulated and discouraged, as was suggested would be necessary over a decade ago [31]. This is being strongly resisted by food and related transnational industries [59,60], despite some encouraging changes in salt-reduced foods and some moves to smaller sizes and helpings. Nevertheless, public health sector and food industry interactions are still very inadequate and relatively ineffective [45].

It will also be necessary to work more towards alleviating health and nutrition care inequities. Health inequalities—the inequitable distribution between the rich and poor of disease prevalence, differential access to healthcare and differing early death rates—have persisted despite substantially increased resources and innovative policies [60]. One solution that is being suggested as part of the post-MDG era is that a framework convention on global health would include, amongst other modalities, “defining national responsibilities for the population’s health; defining international responsibilities for reliable, sustaining funding; setting global health priorities; coordinating fragmented activities; reshaping global governance for health” (including strengthening the WHO) [60]. The WHO, at the direction of the WHA, has launched a commission on childhood obesity. Similarly, the World Cancer Research Fund International has identified country experience in improving healthy eating in areas, such as food labelling, marketing and advertising, taxes and subsidies, food supplies and the food chains and increasing public awareness [61]. All of these are likely to be necessary as complementary to improve and more widely spread healthcare and increased attention to nutrition.

## 6. Conclusions

Recognition of the role of optimal nutrition in both preventive care and as part of good healthcare is receiving increased attention, in the scientific literature, in health-care training and in the lay press. Changing nutrition and dietary patterns are contributing, along with other social and environmental factors, to the current global epidemic of NCD. At the same time, nutrition insecurity still affects over 800 million people globally, and infant, young children and maternal under-nutrition remain widespread, despite successes over the last decade and a half. In 2011, an estimated 101 million children under five years of age globally were still underweight, down from an estimated 159 million in 1990. Contributing to the complex picture of causation and other influencing factors are changed activity patterns, genetic and early foetal origin and early developmental factors of adult diseases, as well as an increasingly global obesogenic environment. The burden of these diseases is increasingly shifting to LMIC, and it is estimated that by 2020, NCD will cause seven out of every 10 deaths and account for 80% of the global burden of disease. This will have implications for healthcare and the distribution and capacity of caregivers. Many countries are suffering from a double burden of both under-nutrition and NCD, and they will be particularly hard hit, further straining already overtaxed health systems, often with already poor capacity. It has been noted by the WHO Commission on Social Determinants of Health that “the development of a society, rich or poor, can be judged by the quality of its population’s health, how fairly health is distributed across the social spectrum, and the degree of protection provided from disadvantage as a result of ill-health” [2].

Costs of nutritional-related NCD and risk factors that constitute metabolic syndrome are predicted to continue to increase and will be an increasing healthcare financial burden, especially in LMIC. Not only are healthcare costs greatly increased by hospitalization due to nutritional-related NCDs, such as type 2 diabetes mellitus and CVD, but also each individual metabolic syndrome component has been found to be associated with higher future medical costs [16]. In countries where healthcare costs are already insufficient, as in much of South Asia and Sub-Saharan Africa, this represents a real threat to continuing improved outcomes of populations.

Nevertheless, as WHO has also pointed out, much of the improved life expectancy in high income countries has been due to better treatment of these diseases, underlying the importance of healthcare and also the challenges [12]. There is a need to invest more heavily, and wisely, in health and nutrition care as, despite progress, “hard choices remain” to be made [62]. All of the above emphasizes the continued need for improved healthcare with accessibility to better health and nutrition services. Increased attention to nutrition during training should continue to be upgraded. Government policies on nutrition interventions, both in healthcare and for more effective public health nutrition interventions, will need to be complementary to address the challenges to and opportunities for scaling-up nutrition in healthcare.
